# Integrated genome-wide investigations of the housefly, a global vector of diseases reveal unique dispersal patterns and bacterial communities across farms

**DOI:** 10.1186/s12864-020-6445-z

**Published:** 2020-01-21

**Authors:** Simon Bahrndorff, Aritz Ruiz-González, Nadieh de Jonge, Jeppe Lund Nielsen, Henrik Skovgård, Cino Pertoldi

**Affiliations:** 1 Department of Chemistry and Bioscience, Section of Biology and Environmental Science, Aalborg University, Fredrik Bajers Vej 7H, DK-9220 Aalborg East, Denmark; 2 Department of Zoology and Animal Cell Biology, University of the Basque Country UPV/EHU, Vitoria-Gasteiz, Spain; 3 Systematics, Biogeography and Population Dynamics Research Group, Lascaray Research Center, University of the Basque Country (UPV/EHU), Vitoria-Gasteiz, Spain; 4 Department of Agroecology, University of Aarhus, DK-4200 Slagelse, Denmark; 5 Aalborg Zoo, DK-9000 Aalborg, Denmark

**Keywords:** Vector, *Musca domestica*, Housefly, Genotyping-by-sequencing, SNPs, Population structure, Microbiome, Pathogens, Isolation by distance

## Abstract

**Background:**

Houseflies (*Musca domestica* L.) live in intimate association with numerous microorganisms and is a vector of human pathogens. In temperate areas, houseflies will overwinter in environments constructed by humans and recolonize surrounding areas in early summer. However, the dispersal patterns and associated bacteria across season and location are unclear. We used genotyping-by-sequencing (GBS) for the simultaneous identification and genotyping of thousands of Single Nucleotide Polymorphisms (SNPs) to establish dispersal patterns of houseflies across farms. Secondly, we used 16S rRNA gene amplicon sequencing to establish the variation and association between bacterial communities and the housefly across farms.

**Results:**

Using GBS we identified 18,000 SNPs across 400 individuals sampled within and between 11 dairy farms in Denmark. There was evidence for sub-structuring of Danish housefly populations and with genetic structure that differed across season and sex. Further, there was a strong isolation by distance (IBD) effect, but with large variation suggesting that other hidden geographic barriers are important. Large individual variations were observed in the community structure of the microbiome and it was found to be dependent on location, sex, and collection time. Furthermore, the relative prevalence of putative pathogens was highly dependent on location and collection time.

**Conclusion:**

We were able to identify SNPs for the determination of the spatiotemporal housefly genetic structure, and to establish the variation and association between bacterial communities and the housefly across farms using novel next-generation sequencing (NGS) techniques. These results are important for disease prevention given the fine-scale population structure and IBD for the housefly, and that individual houseflies carry location specific bacteria including putative pathogens.

## Background

The housefly is a cosmopolitan species and lives in close association with humans. It breeds in animal manure, human excrement, garbage, animal bedding, and decaying organic matter where bacteria are abundant [1]. It is therefore not surprising that the housefly is a well-known carrier of many disease causing microorganisms, including bacteria, virus, fungi, and parasites [1]. The housefly has been found as a carrier of pathogenic bacteria such as *Salmonella* spp. [2], *Shigella* spp. [3], *Campylobacter* spp. [4], *Staphylococcus aureus* [5], *Pseudomonas aeruginosa* [5], *Enterococcus faecalis* [5], and *Escherichia coli* [6]. Furthermore, results have shown the housefly to be an important vector of human pathogens such as *Campylobacter* spp. and *Shigella* spp. [3, 7]. However, dispersal patterns of the housefly and the variation and association of bacteria with the housefly across locations are unclear [8].

Houseflies occur both in the tropics and temperate areas, even though temperature in temperate regions in winter will go below thermal tolerance limits of the species [9]. Houseflies in temperate areas will overwinter in environments constructed by humans, such as poultry barns or with other domestic animals. In spring, populations will increase in numbers, and when outdoor temperatures become permissive, flies will migrate to repopulate the surrounding landscape [10]. Therefore, environmental factors play an important role for local survival and reproduction [11], and are thus also likely to affect the gene flow and selective pressures that the species experience. Genetic differentiation may exist if gene flow is overcome by genetic drift or by local selective pressures [12]. In temperate regions results have shown a strong seasonal change in number of *M. domestica*, which is also one of the species most often carrying *Campylobacter* spp. [13]. This seasonal trend is also present for number of *Campylobacter* spp. positive broiler chicken flocks, but not in broiler chicken houses with fly-screens [7]. Together, this seasonality suggests that dispersal patterns of houseflies could play a key role in obtaining a better understanding of the epidemiology of human pathogens including *Campylobacter*.

A variety of methods have previously been used to evaluate dispersal patterns of houseflies. Mark-release-recapture techniques have been used to estimate dispersal and flight range under natural conditions [14], whereas behavioural patterns have been estimated under laboratory conditions [11]. Population genetic studies have been conducted to estimate gene flow using either microsatellites [15–18] or mitochondrial DNA markers [19–23]. Most studies have evaluated population genetic structure at the macrogeographical level, among continents or regions [17, 18, 20, 23], whereas few studies have addressed micro-geographical genetic structure [15, 16, 21].

Advances in next generations sequencing (NGS) technologies have revolutionized biological sciences including epidemiology and the study of disease vectors. For example, the analysis of environmental DNA through the use of specific gene markers such as species-specific DNA barcodes has been a key application of next-generation sequencing technologies [24, 25]. Such developments could potentially allow the simultaneous study of both the vector species in question together with its associated bacterial community.

To obtain a more detailed and comprehensive genetic data of the housefly population structure and dispersal patterns, we optimized the genotyping-by-sequencing (GBS) protocol for the house fly and genotyped on average twenty individuals from 11 Danish dairy farms across seasons (early and late summer). Genotyping-by-sequencing allow the simultaneous identification and genotyping of thousands of Single Nucleotide Polymorphisms (SNPs) in a large number of samples and is one of the simplest reduced genome representation approaches developed so far [26, 27]. Large panels of SNP markers allow inferring the population genetic structure even at microgeographic level, evidencing the evolutionary processes [28]. SNP markers obtained through GBS and other similar procedures implemented in next-generation sequencing platforms, are widely used to estimate genome-wide diversity in populations of non-model organisms [29, 30], but has not so far been applied in epidemiological studies. We aimed to infer population structure and gene-flow among housefly populations on a local and regional scale. In particular, we were interested in inferring if population structures and gene flow differed between sexes and seasons across farms. Moreover, we investigated if there is evidence of isolation-by-distance (IBD) in Danish populations of the house fly as this can have important implications for the spread of pathogenic bacteria between farms. Secondly, in order to better understand the variation and association between bacteria and the housefly across farms, we used 16S rRNA gene amplicon sequencing to describe bacterial communities.

## Results

### SNP data quality and coverage

The GBS pipeline recovered 1,997,747 putative SNP loci. After stringent filtering based on coverage and presence in the 400 individuals, 18,287 loci SNPs had a sufficiently high quality for downstream genetic analysis, with an overall call rate of 92.97%. Across all loci, the mean coverage per locus per individual was 37.80 (max mean coverage was 96.73 and minimum mean 10.48).

### Genetic variability

The genetic parameters H_O_, H_E,_ and F_IS_ are listed in Table 1. There were significant deviations from Hardy Weinberg Equilibrium (HWE) for all 11 populations investigated (*P* < 0.001). The positive F_IS_ values indicate that deviations from HWE are due to heterozygote deficiency (Table 1).

**Table 1:** Summary of population genetic data from each of 11 populations of the housefly (*Musca domestica*) collected throughout Denmark. Individuals were collected in early summer (1) and late summer (2) and sorted into males and females. The mean and 95% confidence interval of observed heterozygosity (H_O_), expected heterozygosity (H_E_), inbreeding coefficient (F_IS_) is presented

Population	n	Time	Sex	H_O_		H_E_		F_IS_	
				Mean	95% CI	Mean	95% CI	Mean	95% CI
1	9	1	Male	0.242	(0.239–0.244)	0.263	(0.260–0.265)	0.080	(0.073–0.087)
2	10	1	Male	0.247	(0.244–0.249)	0.264	(0.262–0.267)	0.066	(0.060–0.073)
3	9	1	Male	0.245	(0.241–0.247)	0.259	(0.255–0.260)	0.053	(0.046–0.061)
4	9	1	Male	0.250	(0.2470.253)	0.259	(0.256–0.261)	0.035	(0.028–0.043)
5		1	Male	^a^		^a^		^a^	
6	10	1	Male	0.249	(0.246–0.252)	0.268	(0.266–0.271)	0.072	(0.066–0.079)
8	10	1	Male	0.250	(0.247–0.252)	0.268	(0.266–0.271)	0.069	(0.063–0.075)
9	9	1	Male	0.249	(0.246–0.252)	0.266	(0.264–0.269)	0.063	(0.057–0.070)
10	10	1	Male	0.247	(0.245–0.250)	0.266	(0.264–0.268)	0.070	(0.0640.076)
11	10	1	Male	0.247	(0.244–0.250)	0.266	(0.263–0.268)	0.070	(0.064–0.077)
12	10	1	Male	0.248	(0.245–0.250)	0.264	(0.262–0.267)	0.063	(0.056–0-069)
1	7	1	Female	0.236	(0.233–0.239)	0.256	(0.254–0.259)	0.079	(0.071–0.086)
2	10	1	Female	0.238	(0.235–0.240)	0.262	(0.259–0.264)	0.092	(0.085–0.098)
3	9	1	Female	0.242	(0.239–0.245)	0.259	(0.256–0.261)	0.065	(0.058–0.072)
4	10	1	Female	0.241	(0.237–0.243)	0.257	(0.255–0.260)	0.065	(0.059–0.072)
5		1	Female	^a^		^a^			
6	3	1	Female	0.255	(0.241–0.249)	0.269	(0.255–0.262)	0.052	(0.040–0.064)
8	9	1	Female	0.246	(0.243–0.249)	0.265	(0.262–0.267)	0.070	(0.063–0.077)
9	10	1	Female	0.245	(0.242–0.248)	0.264	(0.262–0.267)	0.074	(0.067–0.080)
10	10	1	Female	0.244	(0.242–0.247)	0.264	(0.261–0.266)	0.074	(0.067–0.081)
11	10	1	Female	0.247	(0.244–0.249)	0.265	(0.262–0.267)	0.069	(0.063–0.075)
12	10	1	Female	0.271	(0.2680.274)	0.272	(0.270–0.275)	0.006	(0.000–0.012)
1	10	2	Male	0.244	(0.241–0.246)	0.263	(0.241–0.246)	0.073	(0.067–0.080)
2	9	2	Male	0.245	(0.242–0.248)	0.265	(0.262–0.267)	0.075	(0.068–0-081)
3	9	2	Male	0.245	(0.242–0.248)	0.260	(0.258–0.263)	0.058	(0.051–0.065)
4	9	2	Male	0.253	(0.250–0.256)	0.266	(0.263–0.268)	0.049	(0.043–0.056)
5	10	2	Male	0.242	(0.240–0.245)	0.263	(0.261–0.265)	0.078	(0.072–0-085)
6	10	2	Male	0.246	(0.244–0.249)	0.266	(0.264–0.269)	0.075	(0.069–0.081)
8	10	2	Male	0.246	(0.243–0.249)	0.267	(0.265–0.270)	0.080	(0.073–0.086)
9	10	2	Male	0.248	(0.245–0.251)	0.267	(0.265–0.270)	0.071	(0.065–0.078)
10	10	2	Male	0.245	(0.2420.248)	0.267	(0.264–0.269)	0.082	(0.076–0.089)
11	10	2	Male	0.248	(0.245–0.251)	0.266	(0.264–0.269)	0.069	(0.063–0.075)
12	10	2	Male	0.243	(0.241–0.246)	0.267	(0.265–0.270)	0.089	(0.083–0.095)
1	10	2	Female	0.239	(0.237–0.242)	0.261	(0.259–0.264)	0.083	(0.077–0.089)
2	10	2	Female	0.239	(0.237–0.242)	0.263	(0.260–0.265)	0.089	(0.082–0.095)
3	10	2	Female	0.241	(0.238–0.244)	0.257	(0.255–0.260)	0.063	(0.056–0.069)
4	9	2	Female	0.240	(0.237–0.243)	0.259	(0.256–0.261)	0.073	(0.067–0.080)
5	10	2	Female	0.241	(0.238–0.244)	0.262	(0.260–0.265)	0.080	(0.074–0.087)
6	10	2	Female	0.242	(0.239–0.244)	0.264	(0.262–0.266)	0.084	(0.078–0.091)
8	10	2	Female	0.245	(0.242–0.247)	0.265	(0.263–0.268)	0.077	(0.071–0.083)
9	10	2	Female	0.242	(0.239–0.244)	0.266	(0.264–0.269)	0.092	(0.085–0.098)
10	10	2	Female	0.244	(0.241–0.247)	0.264	(0.261–0.266)	0.075	(0.069–0.081)
11	10	2	Female	0.245	(0.242–0.248)	0.266	(0.263–0.268)	0.078	(0.071–0.084)
12	10	2	Female	0.237	(0.235–0.240)	0.263	(0.260–0.265)	0.097	(0.091–0.104)

^a^ indicates that flies were lacking for these populations. *n* number of individuals included

The genetic divergence between populations ranged from 0 to 0.027 for males and females collected in early summer. In late summer genetic divergence ranged from − 0.002 to 0.019 (Table 2)

**Table 2:** Pairwise F_ST_ values between all the 11 populations investigated for males and females collected in early summer and late summer respectively. All the F_ST_ comparisons were highly significant (*p* < 0.0001)

Population	1	2	3	4	5	6	8	9	10	11	12
Males, early summer											
1	–										
2	0.010	–									
3	0.013	0.019	–								
4	0.015	0.017	0.021	–							
5	^a^	^a^	^a^	^a^	–						
6	0.010	0.007	0.015	0.013	^a^	–					
8	0.008	0.005	0.017	0.017	^a^	0.001	–				
9	0.014	0.012	0.020	0.017	^a^	0.000	0.003	–			
10	0.011	0.009	0.021	0.020	^a^	0.006	0.003	0.007	–		
11	0.009	0.007	0.017	0.017	^a^	0.002	0.000	0.006	0.002	–	
12	0.012	0.011	0.019	0.020	^a^	0.008	0.005	0.012	0.007	0.005	–
Females, early summer											
1	–										
2	0.018	–									
3	0.027	0.018	–								
4	0.026	0.015	0.021	–							
5	^a^	^a^	^a^	^a^	–						
6	0.024	0.008	0.020	0.019	^a^	–					
8	0.020	0.007	0.018	0.017	^a^	0.004	–				
9	0.022	0.008	0.022	0.022	^a^	0.003	0.006	–			
10	0.020	0.003	0.018	0.015	^a^	0.002	0.005	0.003	–		
11	0.020	0.004	0.019	0.017	^a^	0.007	0.005	0.002	0.000	–	
12	0.023	0.008	0.019	0.017	^a^	0.006	0.007	0.009	0.004	0.006	–
Males, late summer											
1	–										
2	0.007	–									
3	0.010	0.012	–								
4	0.008	0.008	0.013	–							
5	0.010	0.004	0.015	0.009	–						
6	0.010	0.004	0.015	0.009	0.004	–					
8	0.010	0.002	0.015	0.010	0.004	0.001	–				
9	0.010	0.004	0.015	0.009	0.004	0.001	0.001	–			
10	0.010	0.006	0.017	0.010	0.007	0.002	0.004	0.003	–		
11	0.010	0.003	0.015	0.011	0.003	0.000	0.002	0.002	0.003	–	
12	0.010	0.007	0.017	0.012	0.005	0.006	0.006	0.007	0.007	0.006	–
Females, late summer											
1	–										
2	0.007	–									
3	0.011	0.015	–								
4	0.010	0.011	0.019	–							
5	0.010	0.005	0.019	0.014	–						
6	0.009	0.006	0.017	0.012	0.003	–					
8	0.009	0.004	0.019	0.012	0.006	−0.001	–				
9	0.010	0.004	0.019	0.011	0.005	−0.002	−0.002	–			
10	0.010	0.006	0.018	0.012	0.003	0.001	0.002	0.002	–		
11	0.008	0.005	0.017	0.013	0.004	0.001	0.000	−0.001	0.000	–	
12	0.011	0.010	0.019	0.016	0.005	0.006	0.007	0.007	0.007	0.006	–

^a^indicates that flies were lacking for these populations

### Population genetic structure

**Fig. 1:**
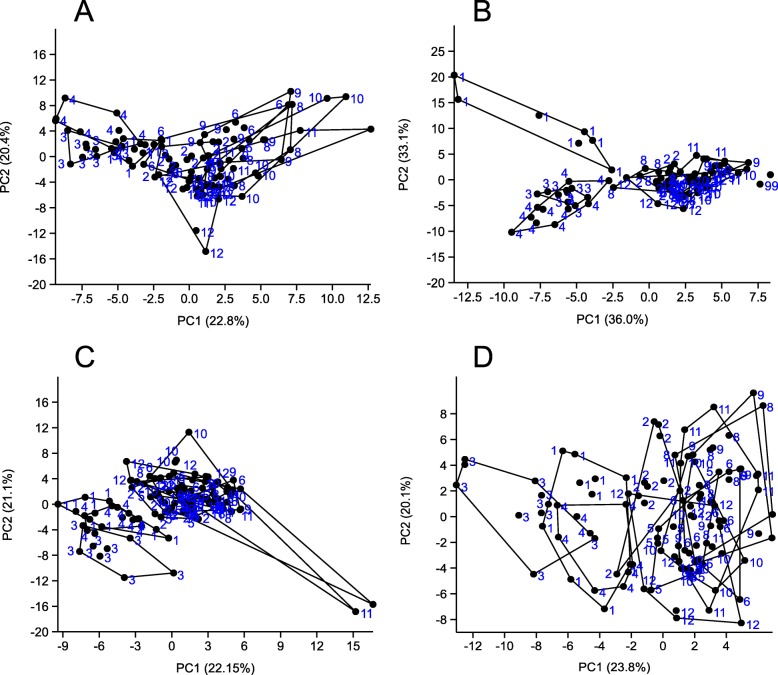
Principal coordinates analysis of SNPs. Plots of the values of the first two components for males collected in early summer (**a**), females collected in early summer (**b**), males collected in late summer (**c**), and females collected in late summer (**d**). Numbers indicate the farms from which individual flies were collected and numbered as in Table 1

There was evidence of population genetic structure among the sampled populations (Fig. 1 ). For males collected in early summer the first two axes of the Principal Component Analysis (PCA) explained 22.8 and 20.4% of the variation, respectively (Fig. 1a). Two distinct clusters were clearly separated by PC1, where the first group includes populations from the eastern study area (i.e. 1, 3, and 4) and the second group includes the remaining populations. For females collected in early summer the first two axes explained 36.0 and 33.1% of the variation, respectively (Fig. 1b). Three distinct clusters were clearly separated by PC1 and PC2, where the first group includes only population 1, the second group includes populations 3 and 4, and the third group includes populations 2, 5, 6, 8, 9, 10, 11 and 12. For males collected in late summer the first two axes explained 22.1 and 22.1% of the variation, respectively (Fig. 1c). Two distinct clusters were clearly separated by PC1 and PC2, where the first group includes populations 1, 3, and 4 and the second group includes the remaining populations. The same pattern was seen for females collected in late summer, where the first two axes explained 23.8 and 20.1% of the variation, respectively (Fig. 1d).

**Fig. 2:**
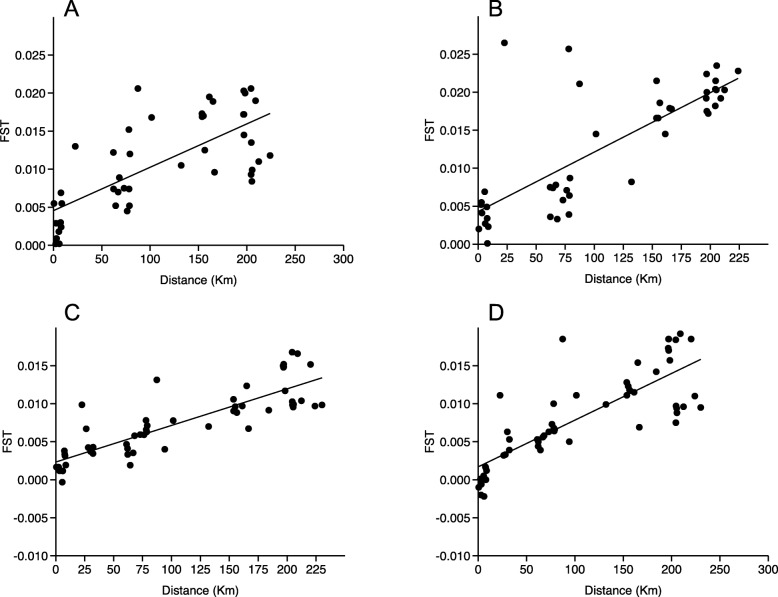
Least square regression of the geographic distance versus the genetic distance (Mantel test) of males collected in early summer (**a**) (slope: 5.70E-05, R^2^ = 0.51, *p* < 0.0001), females collected in early summer (**b**) (slope: 7.82E-05, R^2^ = 0.60, p < 0.0001), males collected in late summer (**c**) (slope: 4.81E-05, R^2^ = 0.71, p < 0.0001), and females collected in late summer (**d**) (slope: 6.14E-05, R^2^ = 0.65, p < 0.0001)

The Mantel tests were performed to test for IBD on all the populations and across season and sex. The regression of the distance between F_ST_ and geographic distance in km was highly significant for all population comparisons (males collected in early summer, R^2^ = 0.51, *p* < 0.0001; females collected in early summer, R^2^ = 0.60, p < 0.0001; males collected in late summer R^2^ = 0.71, p < 0.0001; females collected in late summer R^2^ = 0.65, *p* < 0.0001) (Fig. 2a-d).

The slopes of the regression were found to be higher in early summer for females (slope: 7.82E-05) versus males (slope: 5.70E-05), however, the difference was not significant (F = 2.71, *p* = 0.10). The slopes of the regression were also found to be higher in late summer for females (slope: 6.14E-05) versus males (slope: 4.81E-05), however, also in this case, the difference was not significant (F = 3.20, *p* = 0.07).

The comparisons of males collected in early summer versus males collected in late summer showed a higher slope in early summer compared to late summer, but the difference was not significant (F = 0.99, *p* = 0.32). The same tendency was found for females, but also in this case the difference was not significant F = 2.29, *p* = 0.13).

The minimum cross-validation error (CVE) in the ADMIXTURE analysis suggested optima for K = 2 when including both timepoints and sexes (Additional file 1: Figure S1). Here individuals from eastern sampling populations (1, 3, and 4) were assigned to cluster K1 and the remaining populations were assigned to cluster K2. However, when analysing separately, males and females collected in early summer or late summer, the ADMIXTURE analyses failed to find genetic structure as all the optima were K = 1 (Additional file 2: Figure S2).

### Diversity of bacterial communities within and between locations

A total of 455 samples were sequenced using 16S rRNA gene sequencing of the V1-V3 hypervariable region. The microbiome analysis yielded a grand total of 9,936,255 reads at an average 21,838 ± 9644 reads per sample. A total of 11,482 OTUs were identified.

Based on the rarefaction curve, 5000 reads was selected as the minimum criterion for inclusion in further analysis, which removed 23 samples that did not meet the requirement. Subsequently, a total of 432 samples entered the analysis.

Biodiversity was assessed using a rank abundance curve (Additional file 3: Figure S3), which showed that at least 80% of the total reads per location was associated to the 100 most abundant OTUs.

**Fig. 3:**
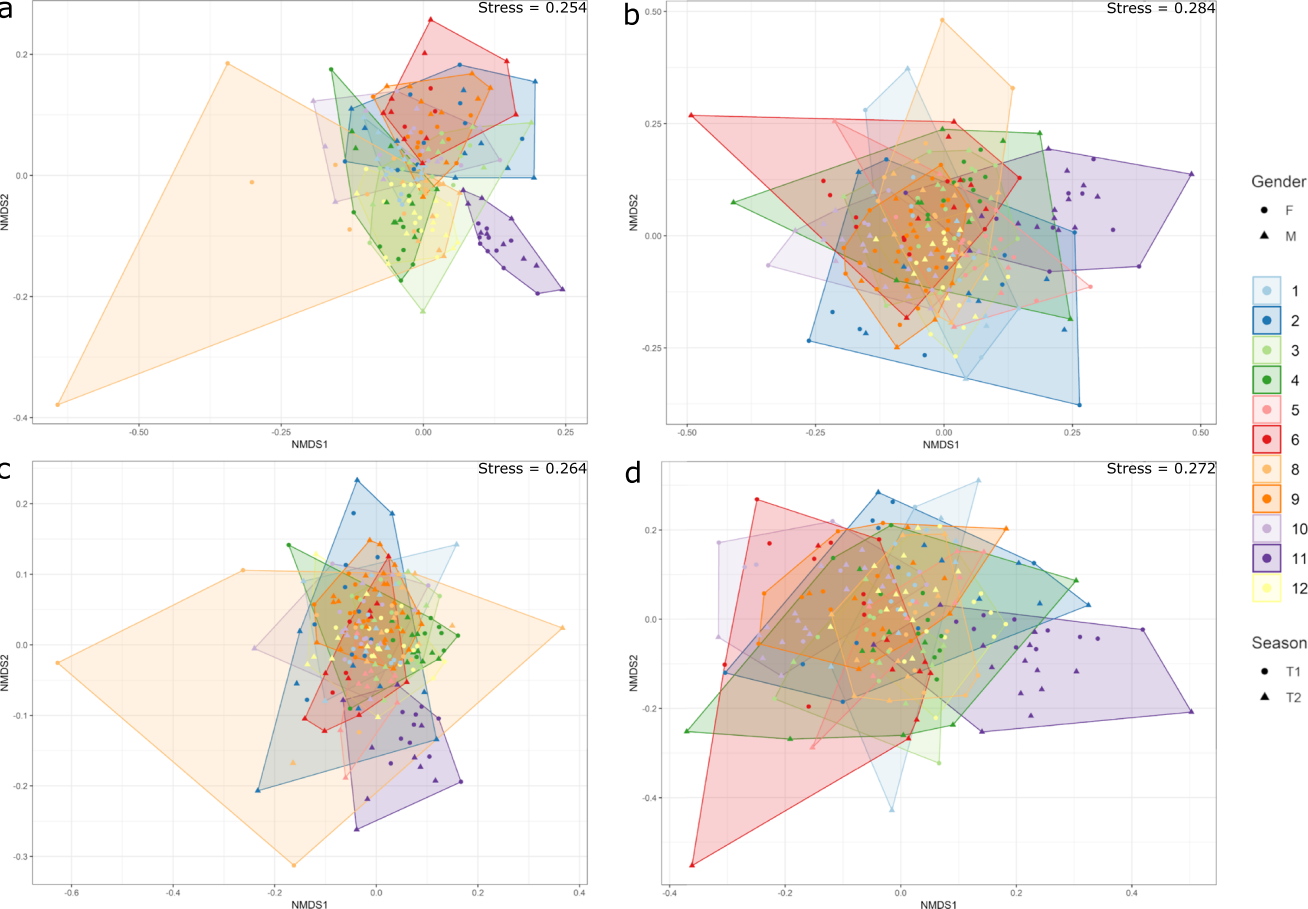
Beta diversity of 11 housefly populations. Non-metric multi-dimensional scaling analysis of houseflies collected in early summer (**a**), late summer (**b**), females (**c**), and males (**d**). Samples are colored by population, and those representing the same population are surrounded by a polygon

Nonmetric multidimensional scaling (NMDS) ordination was used to visualize differences in the community structure between collection time and sex (Fig. 3a–d). There was some separation between locations in bacterial communities. For example, location 10 differed from the remaining locations in early summer, whereas this difference was not evident in late summer. The stress value is lowest for housefly populations collected in early summer (0.254) and highest for populations collected in late summer (0.284).

Statistical testing using PERMANOVA showed that location, sex and collection time all represented significant differences (*p* < 0.001) in the microbial community composition. However, these differences only explained a small portion of the generated model, with R^2^ = 0.142 for location, R^2^ = 0.006 for sex and R^2^ = 0.014 for collection time.

### Bacterial community composition

The bacterial taxa associated with the houseflies collected across sites showed that the microbiome was dominated by the orders, *Lactobacillales*, *Corynebacteriales*, *Clostridiales*, *Flavobacteriales*, *Rhizobiales,* and *Micrococcales* (data not shown). The most abundant OTUs included *Corynebacterium variabile, Vagococcus, Corynebacterium xerosis, Staphylococcus equorum,* and *Lactococcus* (Additional file 4: Figure S4). Furthermore, for some of the abundantly observed OTUs, such as *Acetobacter* and *Lactobacillus sililis,* relative abundance largely varied across season and sex.

**Fig. 4:**
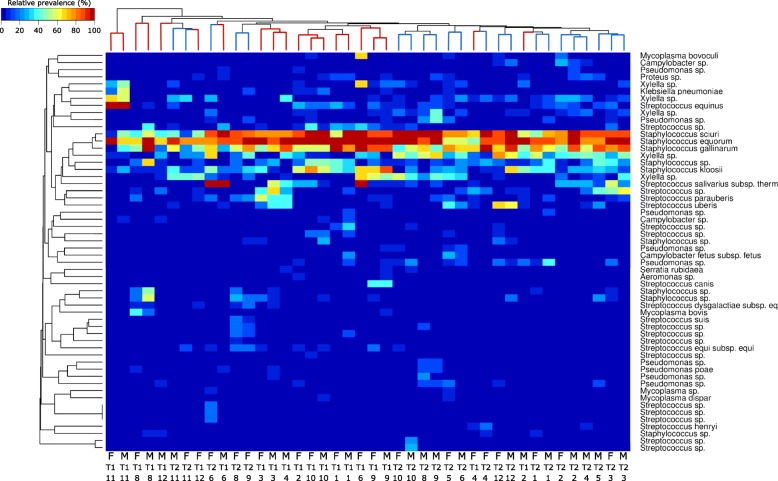
Hierarchially clustered heatmap of relative potential pathogen prevalence. Organisms were selected using a short list of potential pathogenic organisms associated with houseflies. Only organisms that were observed as 0.1% or more of total reads in more than one sample were included. The samples and species were clustered using Bray-Curtis distances, and the sample dendrogram is colored by collection time

A hierarchically clustered heatmap of the relative prevalence of potential pathogens showed that some OTUs were present across most sites and with a relatively high abundance and included, for example, *Staphylococcus sciuri*, *Staphylococcus equorum,* and *Staphylococcus gallinarum* (Fig. 4). Contrary to this pattern, some OTUs were present with low relative prevalence across all sites and included for example *Mycoplasma dispar* and *Campylobacter fetus*. Lastly, some species were present with higher relative prevalence and their presence seemed to be dependent on location and collection time. Species with this pattern included *Streptococcus equinus, Klebsiella pneumoniae, Xylella* sp.*, Mycoplasma bovoculi, Staphylococcus* sp*.,* and *Streptococcus* sp. Overall, the prevalence of potential pathogens showed a trend towards dependency on collection time.

## Discussion

In temperate areas, the housefly has been shown as a carrier and major vector of human pathogens such as *Campylobacter* spp. [7]. Therefore, the identification of important dispersal routes has the potential to mitigate the spread of pathogens by houseflies. Data to elucidate dispersal and populations structure has mainly been obtained at a regional level or across continents. To the best of our knowledge, this investigation is the first to apply novel NGS techniques to simultaneously a) identify SNPs for the determination of the spatiotemporal housefly genetic structure and b) to establish the variation and association between bacterial communities and the housefly across farms.

In the present study, we identified 18,287 SNPs across 400 individuals collected across 11 farms. We found significant deviations from HWE for all 11 farms investigated with positive F_IS_ values due to heterozygosity deficiency, which suggest further sub-structuring of Danish housefly populations and/or strong dynamics and population fluctuations during the period of collection [31]. These results are also supported by the PCA plots showing a weak genetic structure across season and sex. Two main genetic groups where identified, indicating a subdivision of eastern farm populations and the remaining populations. Only for females collected in early summer is there an additional subdivision of group 1, where population 1 is clustering separately from the other eastern population (i.e populations 3 and 4). *Musca domestica* will in temperate areas overwinter in environments constructed by humans, such as poultry barns or with other domestic animals. In spring populations will again increase in numbers and when reaching a certain threshold it will migrate to repopulate the surrounding landscape [9]. These seasonal population dynamics may explain the observed substructuring.

The lack of a strong genetic structure did not allow ADMIXTURE to find optima above K = 2, which is likely due to a dynamic dispersal process of the houseflies. Dispersal seems to occur via a stepping stone model, creating a clear IBD pattern. This is supported by the significant correlations for genetic and geographic distances. It is also noteworthy that despite the fact that the differences between sexes in the slopes in both periods of collection are not significant, there is a clear tendency for higher slopes in females compared to males, which indicates a lower dispersal capacity of the females compared to males as has also been found for locomotor activity [32]. Furthermore, the slopes of the regressions in the Mantel tests are higher in early summer compared to late summer for both sexes. This supports the contention that dispersal changes with season as flies are found at lower densities in early summer compared to late summer, where flies are found at high densities, suggesting that the populations are more admixed in late summer compared to early summer. This is also supported by the fact that the genetic divergence among populations were found to be higher in early summer (range from 0 to 0.027 for males and females), compared to the genetic divergences observed in late summer (range from − 0.002 to 0.019 for males and females).

The relatively high heterogeneity observed in the distances of the residuals of the regression for the IBD lines, suggest that an IBD exists, but that the genetic distances between populations can also be high despite varying geographic distances between populations. This pattern demonstrates a quite chaotic scenario where hidden geographic barriers must exist or where high gene flow between geographically separated populations is found (due to random transport of flies by vehicles, for example). Understanding dispersal patterns can thus help us develop strategies to reduce the number of infectious diseases, such as campylobacteriosis [7]. In addition, dispersal of flies across large distances may also help explain disease outbreaks in other systems, such as African swine fever in temperate areas, where transmission routes are not fully [33, 34].

The present study provides an in-depth analysis of the adult housefly microbiome on an unprecedently large number of single whole flies on a regional and microscale, and in combination with a detailed SNP dataset. We were able to identify multiple species of bacteria that in earlier studies have been associated with filth flies and suggested or established as potential pathogenic bacteria [4, 5, 35–38]. It is also evident that some bacterial species, being potential human pathogens, are only present in single flies or farms, whereas other species are present across all sites and on most flies (Fig. 4). Comparing with published housefly microbiomes, genera such as *Weissella*, *Staphylococcus,* and *Corynebacterium* are consistently present in adult houseflies [39, 40]. The microbiome of flies of the respective sites and timepoints generally group together (Fig. 4), and there also seems to be a trend in that stronger differentiation exists in early summer (Fig. 3).

## Conclusions

In conclusion, the results of the present study detected a distribution of potential human pathogens dictated by locations and season, and genetic structure dominated by IBD across sex and season. These results highlight that the microbiota of putative pathogenic bacteria in houseflies are dependent on location and that dispersal from locations are more likely to happen on a local scale and in late summer. This highlights the potential for flies as carriers or vectors of human pathogens and importance under temperate conditions for disease transmission.

The study highlights the usefulness of next generation sequencing for epidemiological studies and allowed not only the SNPs of single flies to be established, but also the microbiome of the same individual. The data set provided by the NGS techniques could pave the way for modelling scenarios where the probability of dispersal of flies could be put in a context of probability of diseases transmission.

## Methods

### Sample collection

**Fig. 5:**
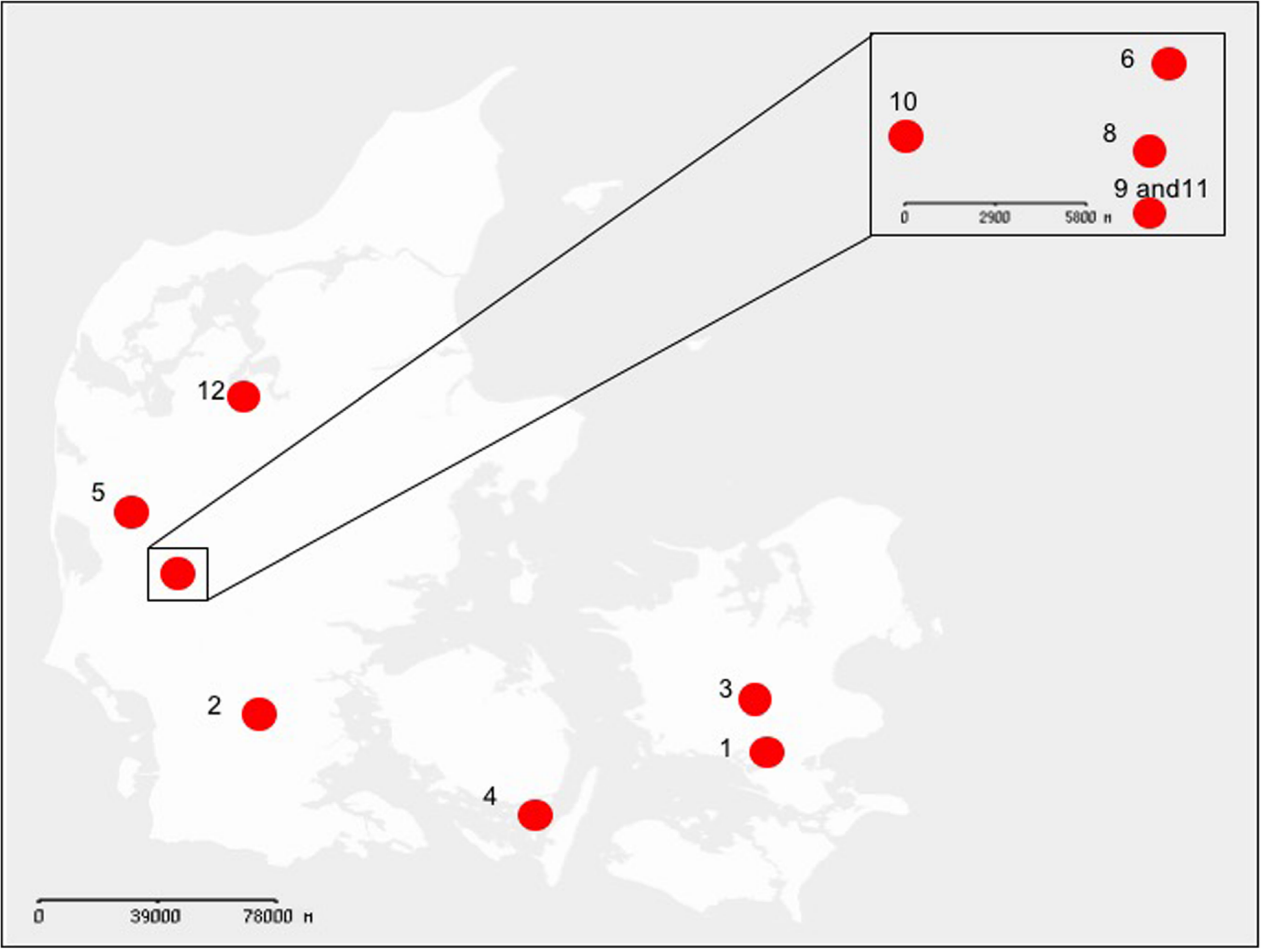
Map of the zone of collections. Sample locations are designated with a number. In total flies from 11 locations (dairy farms) were sampled throughout Denmark. A GeoDanmark Basis map was downloaded from the Danish “Geodatastyrelsen”, October 2016, Styrelsen for Dataforsyning og Effektivisering. The figure is similar, but not identical to the original image, and is therefore for illustrative purposes only

Flies were collected at two timepoints and from 11 dairy farms in 2012 using a sweeping net (Fig. 5). First collection of flies was on the 22nd of June 2012 (hereafter “early summer”) and the second collection was on the 6th of September 2012 (hereafter “late summer”). Flies were collected from the inside of the farms in closed areas with calves walking on deep litter. The farms were located throughout Denmark and were all farms with similar farming practice and manure management (For further details see [8]). *Musca domestica* species identity of flies was established as described elsewhere [41]. Flies were immediately stored in 99.5% ethanol and kept on ice upon transport to the laboratory and subsequently stored at − 20 °C until further processing.

### DNA samples

Total genomic DNA was extracted from whole bodies of houseflies using DNeasy Blood & Tissue Kit (QIAGEN GmbH, Hilden, Germany) according to the manufacturer’s protocol and following the Qiagen supplementary protocol for purification of total DNA from insects. In short, flies were grinded in liquid nitrogen using a pestle in 1.5 ml microcentrifuge tubes. Subsequently, 180 μl ATL buffer and 20 μl of proteinase K were added and incubated overnight at 56 °C. The day after 4 μl of RNase (100 mg/ml) was added to each sample and incubated for 1 h at room temperature before following standard protocol. DNA from each sample was eluted into 100 μl of AE-buffer and subsequently stored at − 20 °C until further use. DNA concentrations were determined using a fluorometer (Qubit®, Thermo Fisher Scientific Inc.) and DNA quality of each sample was assessed visually by electrophoresis ((100 ng DNA loaded on 1% agarose gel)).

### GBS protocol optimization

To determine the best suited restriction endonuclease for the standard GBS protocol [27] DNA of five flies was pooled to obtain a single DNA sample (500 ng) that was digested for 2 h with three different restriction endonucleases: *Ape*KI, *Eco*T22I and *Pst*I (using a tenfold excess of enzyme), at reaction conditions specified by the endonuclease manufacturer (New England Biolabs® Inc.). The digested house fly DNA fragments were subsequently ligated to appropriate adaptors (fitting to the overhang of the DNA insert, left by the restriction endonuclease) for the subsequent PCR (containing barcode adaptors and a “common” adaptor). Adaptor amounts were determined by titration as recommended [27]. Fragment size distributions of each of the three libraries were visualized on a BioAnalyzer 2100 (Agilent Technologies) (Additional file 5: Figure S5). Based on these results, libraries obtained from the *Ape*KI was selected as the enzyme to digest individual house fly DNAs for GBS library generation.

### Preparation of Illumina libraries for next-generation sequencing

Five 96-plex *Ape*KI GBS libraries were prepared according to Elshire et al. [27], each comprising 90–92 DNA samples and 4–6 negative controls to detect potential contaminations. Individual DNA samples were digested with the restriction enzyme and followed by ligation of adapter pairs. The adapters comprised a set of 96 different barcodes containing adaptors and a “common” adaptor. Individual ligations were then pooled and purified from adaptor excess using the QIAquick PCR Purification Kit (QIAGEN). The genomic fragments were subsequently amplified in 50 μL volumes with containing 2 μL of the DNA fragment pool, 1× Taq Master Mix (New England Biolabs® Inc.), and 25 pmol each of the following Illumina primers:

5′-AATGATACGGCGACCACCGAGATCTACACTCTTTCCCTACACGACGCTCTTCCGATCT-3′ and 5′-CAAGCAGAAGACGGCATACGAGATCGGTCTCGGCATTCCTGCTGAACCGCTCTTCCGATCT-3′. (the underlined parts will hybridize to the two Illumina flowcell oligos).

PCR cycling consisted of 72 °C for 5 min, 98 °C for 30 s followed by 18 cycles of 98 °C for 30 s, 65 °C for 10 s, and 72 °C for 30 s, with a final extension step at 72 °C for 5 min. The *Ape*KI GBS libraries were purified again as described above. An aliquot was run on the BioAnalyzer 2100 to verify fragment sizes and removal of adapter dimers. Library-DNA was then quantified on a Nanodrop 2000 (Thermo Scientific). GBS library preparation, sequencing on an Illumina HiSeq 2000, and SNP calling were performed at the Genomic Diversity Facility (GDF) at Cornell University, Biotechnology Resource Center, USA.

### 16S rRNA gene amplicon sequencing

The V1–3 hypervariable region of the bacterial 16S rRNA gene was amplified using the previously described V1–3 primers set 27F/534R (5′-AGAGTTTGATCCTGGCTCAG-3′/5′-ATTACCGCGGCTGCTGG-3′) [42]. The samples were sequenced in equimolar concentrations on a MiSeq platform (Illumina, USA) using MiSeq reagent kit v3 (2 × 300 PE). For further details see descriptions published elsewhere [8].

### Bioinformatic processing and statistical analysis of SNP data

The raw sequence data were processed through the GBS analysis pipeline, implemented in TASSEL v3.0 [43]. To determine genomic coordinates of the variants, sequence tags were aligned to the available genome assembly of the housefly [44] using the Burrows-Wheeler short read alignment tool [45].

The resulting raw dataset from the reference GBS pipeline (1,997,747 SNPs) was further filtered using Golden Helix SNP, Variation Suite SVS v7.2.2 (Golden Helix, Bozeman, MT) and PLINK v1.07 [46]. To facilitate downstream analyses, bi-allelic SNPs was exclusively retained. The dataset was subsequently filtered by the application of genotype-level filters to remove genotypes with low read depths (RD < 5×) and/or low genotype quality (GQ < 98). SNPs and individuals with call-rates < 85%, all SNPs with a minor allele frequency (MAF) < 0.05 and all SNPs with a mean observed heterozygosity (H_o_) greater than 0.6 (to filter out potential paralogs) were removed. Finally, the dataset was pruned for SNPs at strong linkage disequilibrium (LD r^2^ > 0.7) in a window of 50 SNPs (sliding window overlap 5 SNPs at a time). The stringent filtering left 18,287 loci and about 90% of all individuals for further analysis.

The observed and expected heterozygosity (H_O_ and H_E_) was assessed for each population using the software GenAlEx v6.501 [47]. Confidence intervals (95% CI) were calculated for each genetic parameter. Deviations from Hardy-Weinberg Equilibrium (HWE) and the inbreeding coefficient (F_IS_) were estimated using GENEPOP v4.3 [48].

Principal Component Analysis (PCA) based on codominant genotypic distances was used to visualize the genetic relationship among individuals using GenAlEx v6.501 [47]. Additionally, population pairwise genetic distance (F_ST_) (Weir and Cockerham 1984) were calculated using GENEPOP v4.3 [48] to support the PCA analysis. All population pairwise F_ST_ analyses were significant due to the large number of SNPs compared.

A maximum likelihood-based clustering algorithm implemented in ADMIXTURE v1.23 [49] was applied to the filtered house fly dataset to identify the putative ancestral cluster(s) within the entire sample and to assess the extent of genetic admixture. Clustering was performed 100 times for all K-values from K = 2 to K = 12, and the best-fitting K was selected based on the lowest cross-validation error (CV) in combination with the actual genetic patterns revealed by ADMIXTURE runs [49].

The SNP dataset was analysed for the presence of IBD using the Mantel’s test [50]. Pairwise genetic distances among all individuals were plotted against all geographical distances among the corresponding sampling sites within Denmark (actual positions of the farms). Isolation by distance is a common spatial genetic pattern in mobile and continuously distributed species [51] and its existence may represent a challenge to the performance of clustering methods [52]. The slopes of the regression analyses of males versus females were tested for significant differences analysing individuals collected in early summer separately. In addition, the tests for differences between slopes were also performed by comparing the slope of males collected in early summer versus the slope of males collected in late summer and the slope of females collected in early summer versus the slope of females collected in late summer.

### Bioinformatic processing and statistical analysis of microbiome data

The obtained sequence libraries were subjected to quality control using trimmomatic (v0.32) [53]. Reads were merged using FLASH (v1.2.7) [54]. Reads were formatted for use with the UPARSE workflow [55], prior to chimeric read removal, de-replication and clustering into Operational Taxonomic Units (OTUs) at 97% sequence similarity using USEARCH7. Taxonomy was assigned using RDP classifier [56] as implemented in QIIME [57], using Silva release 132 as the reference database [58].

The statistical analysis and visualization of microbial community data was performed in R version 3.5.1 [59] via RStudio version 1.1.463 (http://www.rstudio.com), using the R packages ampvis2, vegan and ggplot2 [60–62]. Beta diversity was calculated for microbiome comparison between housefly from different locations using Bray-Curtis dissimilarity [63], and visualized using non-metric multi-dimensional scaling (NMDS). The microbial community structure was visualized using heatmaps. Relationships between prevalence of potential pathogenic organisms based on literature and the sampled populations were explored using hierarchical clustering using Bray-Curtis distances.

## Supplementary information

**Additional file 1 **: **Figure S1**. ADMIXTURE cross-validation error results. Plot of ADMIXTURE cross-validation error from K=1 through K=6 for both timepoints and sexes. Analysis with K = 2 gave the lowest cross-validation error.

**Additional file 2 **: **Figure S2**. ADMIXTURE cross-validation error results. Plots of ADMIXTURE cross-validation error from K=1 through K=6 for males collected in early summer (a), females collected in early summer (b), males collected in late summer (c), and females collected in late summer (d). Analysis with k = 1 gave the lowest cross-validation error in all cases.

**Additional file 3 **: **Figure S3**. Rank abundance curve. Rank abundance curve of sequencing data generated using 432 houseflies. A line is drawn at 100 OTUs, representing 80% of total read abundance.

**Additional file 4 **: **Figure S4**. Heatmap of abundantly observed OTUs. Microbial community composition. Heatmap of the 50 most abundantly observed OTUs in 11 sampled populations, sorted by season, then by sex. The highest possible taxonomic classification is displayed for each OUT.

**Additional file 5 **: **Figure S5**. Fragment size distribution. Fragment size distribution of GBS libraries made with a single DNA sample using a) ApeKI; b) EcoT22I; and c) PstI restriction enzymes. The x-axis represents elution time and the y-axis shows fluorescence units. Numbers below hatch marks on the x-axis indicate fragment size (bp). Peaks at 15 and 1500 bp are size standards.

## Data Availability

SNP data of the current study is available at Dryad (10.5061/dryad.0gb5mkkxf). All amplicon data are available at European Nucleotide Archive (ENA) under project numbers PRJEB15078 and PRJEB35827.

## Abbreviations

CVE: Cross-validation error
ENA: European Nucleotide Archive
GBS: Genotyping-by-sequencing
HWE: Hardy Weinberg equilibrium
IBD: Isolation by distance
NGS: Next generations sequencing
NMDS: Nonmetric multidimensional scaling
OTU: Operational taxonomic unit
PCA: Principal component analysis
RDP: Ribosomal Database Project
SNP: Single nucleotide polymorphisms

## References

[1] Greenberg B (1973). Flies and diseases vol II, biology and disease transmission.

[2] Holt PS, Geden CJ, Moore RW, Gast RK (2007). Isolation of *Salmonella enterica* serovar Enteritidis from houseflies (*Musca domestica*) found in rooms containing *Salmonella serovar* Enteritidis-challenged hens. Appl Environ Microbiol.

[3] Cohen D, Green M, Block C, Slepon R, Ambar R, Wasserman SS (1991). Reduction of transmission of shigellosis by control of houseflies (*Musca domestica*). Lancet..

[4] Förster M, Sievert K, Messler S, Klimpel S, Pfeffer K (2009). Comprehensive study on the occurrence and distribution of pathogenic microorganisms carried by synanthropic flies caught at different rural locations in Germany. J Med Entomol.

[5] Fotedar R., Banerjee U., Singh S., Shriniwas, Verma A.K. (1992). The housefly (Musca domestica) as a carrier of pathogenic microorganisms in a hospital environment. Journal of Hospital Infection.

[6] Kobayashi M, Sasaki T, Saito N, Tamura K, Suzuki K, Watanabe H (1999). Houseflies: not simple mechanical vectors of enterohemorrhagic *Escherichia coli*O157:H7. Am J Trop Med Hyg.

[7] Bahrndorff S, Rangstrup-Christensen L, Nordentoft S, Hald B (2013). Foodborne disease prevention and broiler chickens with reduced *Campylobacter* infection. Emerg Infect Dis.

[8] Bahrndorff S, de Jonge N, Skovgård H, Nielsen JL (2017). Bacterial communities associated with houseflies (*Musca domestica* L.) sampled within and between farms. PLoS One.

[9] Goulson D, Derwent LC, Hanley ME, Dunn DW, Abolins SR (2005). Predicting calyptrate fly populations from the weather, and probable consequences of climate change. J Appl Ecol.

[10] Black WC, Krafsur ES (1986). Population biology and genetics of winter house fly (Diptera: Muscidae) populations. Ann Entomol Soc Am.

[11] Kjærsgaard A, Blanckenhorn WU, Pertoldi C, Loeschcke V, Bahrndorff S (2015). Plasticity in behavioural responses and resistance to temperature stress in *Musca domestica*. Anim Behav.

[12] Gandon S, Nuismer SL (2009). Interactions between genetic drift, gene flow, and selection mosaics drive parasite local adaptation. Am Nat.

[13] Hald B, Bang DD, Pedersen K, Dybdahl J, Madsen M, Study T (2004). Flies and campylobacter broiler flocks. Emerg Infect Dis.

[14] Nazni WA, Luke H, Wan Rozita WM, Abdullah AG, Sa’diyah I, Azahari AH (2005). Determination of the flight range and dispersal of the house fly, *Musca domestica* (L.) using mark release recapture technique. Trop Biomed.

[15] Chakrabarti S, Kambhampati S, Zurek L (2010). Assessment of house fly dispersal between rural and urban habitats in Kansas, USA. J Kansas Entomol Soc.

[16] Chakrabarti S, Kambhampati S, Grace T, Zurek L (2004). Characterization of microsatellite loci in the house fly, *Musca domestica* L. (Diptera: Muscidae). Mol Ecol Notes.

[17] Krafsur ES, Cummings MA, Endsley MA, Marquez JG, Nason JD (2005). Geographic differentiation in the house fly estimated by microsatellite and mitochondrial variation. J Hered.

[18] Endsley M, Baker M, Krafsur E (2002). Microsatellite loci in the house fly, *Musca domestica* L. (Diptera: Muscidae). Mol Ecol Notes.

[19] Cummings MA, Krafsur ES (2005). Spatial diversity in mitochondrial cytochrome c oxidase in house flies. Med Vet Entomol.

[20] Marquez JG, Krafsur ES (2002). Gene flow among geographically diverse housefly populations (*Musca domestica* L.): a worldwide survey of mitochondrial diversity. J Hered.

[21] Marquez JG, Moon RD, Krafsur ES (2001). Genetic differentiation among populations of house flies (Diptera: Muscidae) breeding at a multiple-barn, egg-laying facility in Central Minnesota. J Med Entomol.

[22] Marquez JG, Bangs MJ, Krafsur ES (2003). Mitochondrial diversity of *Musca domestica* housefly populations in the Asian and western Pacific biogeographical regions. Med Vet Entomol.

[23] Marquez JG, Krafsur ES (2003). Mitochondrial diversity evaluated by the single strand conformation polymorphism method in African and North American house flies (*Musca domestica* L.). Insect Mol Biol.

[24] Cain AK, Lees JA (2015). Using genomics to combat infectious diseases on a global scale. Genome Biol.

[25] Rinker DC, Pitts RJ, Zwiebel LJ (2016). Disease vectors in the era of next generation sequencing. Genome Biol.

[26] Davey JW, Hohenlohe PA, Etter PD, Boone JQ, Catchen JM, Blaxter ML (2011). Genome-wide genetic marker discovery and genotyping using next-generation sequencing. Nat Rev Genet.

[27] Elshire Robert J., Glaubitz Jeffrey C., Sun Qi, Poland Jesse A., Kawamoto Ken, Buckler Edward S., Mitchell Sharon E. (2011). A Robust, Simple Genotyping-by-Sequencing (GBS) Approach for High Diversity Species. PLoS ONE.

[28] Thirstrup J. P., Ruiz-Gonzalez A., Pujolar J. M., Larsen P. F., Jensen J., Randi E., Zalewski A., Pertoldi C. (2015). Population genetic structure in farm and feral American mink (Neovison vison) inferred from RAD sequencing-generated single nucleotide polymorphisms1. Journal of Animal Science.

[29] Ekblom R, Galindo J (2010). Applications of next generation sequencing in molecular ecology of non-model organisms. Heredity.

[30] Garrick RC, Bonatelli IAS, Hyseni C, Morales A, Pelletier TA, Perez MF (2015). The evolution of phylogeographic data sets. Mol Ecol.

[31] Hartl DL, Clark AG (1989). Principles of population genetics.

[32] Schou TM, Faurby S, Kjærsgaard A, Pertoldi C, Loeschcke V, Hald B, et al. Temperature and population density effects on Locomotor activity of *Musca domestica* (Diptera: Muscidae). Environ Entomol. 2013;42:1322–8. 10.1603/EN13039 24246478

[33] Herm R, Tummeleht L, Jürison M, Vilem A, Viltrop A. Trace amounts of African swine fever virus DNA detected in insects collected from an infected pig farm in Estonia. Vet Med Sci. 2019:1–5. 10.10021/vms3.200 10.1002/vms3.200 PMC7036316 31560174

[34] Olesen Ann Sofie, Lohse Louise, Hansen Mette Frimodt, Boklund Anette, Halasa Tariq, Belsham Graham J., Rasmussen Thomas Bruun, Bøtner Anette, Bødker René (2018). Infection of pigs with African swine fever virus via ingestion of stable flies (Stomoxys calcitrans ). Transboundary and Emerging Diseases.

[35] Banjo AD, Lawal OA, Adeduji OO (2005). Bacteria and fungi isolated from housefly (*Musca domestica* L.) larvae. African J Biotechnol.

[36] Hald B., Skovgård H., Pedersen K., Bunkenborg H. (2008). Influxed Insects as Vectors for Campylobacter jejuni and Campylobacter coli in Danish Broiler Houses. Poultry Science.

[37] Szalanski A. L., Owens C. B., Mckay T., Steelman C. D. (2004). Detection of Campylobacter and Escherichia coli O157:H7 from filth flies by polymerase chain reaction. Medical and Veterinary Entomology.

[38] Förster Maike, Klimpel Sven, Mehlhorn Heinz, Sievert Kai, Messler Sabine, Pfeffer Klaus (2007). Pilot study on synanthropic flies (e.g. Musca, Sarcophaga, Calliphora, Fannia, Lucilia, Stomoxys) as vectors of pathogenic microorganisms. Parasitology Research.

[39] Junqueira ACM, Ratan A, Acerbi E, Drautz-m DI, Premkrishnan BNV, Costea PI (2017). The microbiomes of blowflies and houseflies as bacterial transmission reservoirs. Sci Rep.

[40] Park R, Dzialo MC, Spaepen S, Nsabimana D, Gielens K, Devriese H, et al. Microbial communities of the house fly *Musca domestica* vary with geographical location and habitat. Microbiome. 2019;7:1–12. 10.1186/s40168-019-0748-9 PMC6839111 31699144

[41] Thomsen M. Stuenfluen (*Musca domestica*) og stikfluen (*Stomoxys calcitrans*). Undersøgelser over biologi og bekæmpelse samt en oversigt over andre til husdyr eller boliger knyttede fluearter. Forsøgslaboratoriet. 1938;176.

[42] Ward DV, Gevers D, Giannoukos G, Earl AM, Methé BA, Sodergren E (2012). Evaluation of 16s rDNA-based community profiling for human microbiome research. PLoS One.

[43] Glaubitz JC, Casstevens TM, Lu F, Harriman J, Elshire RJ, Sun Q (2014). TASSEL-GBS: a high capacity genotyping by sequencing analysis pipeline. PLoS One.

[44] Scott JG, Warren WC, Beukeboom LW, Bopp D, Clark AG, Giers SD (2014). Genome of the house fly, *Musca domestica* L., a global vector of diseases with adaptations to a septic environment. Genome Biol.

[45] Li H, Durbin R (2009). Fast and accurate short read alignment with burrows-wheeler transform. Bioinformatics..

[46] Purcell S, Neale B, Todd-Brown K, Thomas L, Ferreira MAR, Bender D (2007). PLINK: a tool set for whole-genome association and population-based linkage analyses. Am J Hum Genet.

[47] Peakall R, Smouse PE (2012). GenALEx 6.5: genetic analysis in excel. Population genetic software for teaching and research-an update. Bioinformatics..

[48] Rousset F (2008). GENEPOP’007: a complete re-implementation of the GENEPOP software for windows and Linux. Mol Ecol Resour.

[49] Alexander DH, Novembre J, Lange K (2009). Fast model-based estimation of ancestry in unrelated individuals. Genome Res.

[50] Mantel N (1967). The detection of disease clustering and a generalized regression approach. Cancer Res.

[51] Wright S (1943). Isolation by distance. Genetics..

[52] Meirmans PG (2012). The trouble with isolation by distance. Mol Ecol.

[53] Bolger AM, Lohse M, Usadel B (2014). Trimmomatic: a flexible trimmer for Illumina sequence data. Bioinformatics..

[54] Magoč T, Salzberg SL (2011). FLASH: fast length adjustment of short reads to improve genome assemblies. Bioinformatics..

[55] Edgar RC (2013). UPARSE: highly accurate OTU sequences from microbial amplicon reads. Nat Methods.

[56] Wang Q, Garrity GM, Tiedje JM, Cole JR (2007). Naive Bayesian classifier for rapid assignment of rRNA sequences into the new bacterial taxonomy. Appl Environ Microbiol.

[57] Caporaso JG, Kuczynski J, Stombaugh J, Bittinger K, Bushman FD, Costello EK (2010). QIIME allows analysis of high- throughput community sequencing data intensity normalization improves color calling in SOLiD sequencing. Nat Methods.

[58] Quast C, Pruesse E, Yilmaz P, Gerken J, Schweer T, Yarza P (2013). The SILVA ribosomal RNA gene database project: improved data processing and web-based tools. Nucleic Acids Res.

[59] R Core Team (2017). R: A language and environment for statistical computing.

[60] Wickham H (2009). ggplot2: elegant graphics for data analysis.

[61] Andersen KS, Kirkegaard RH, Karst SM, Albertsen M. ampvis2: an R package to analyse and visualise 16S rRNA amplicon data. 2018; 10–11. bioRxiv. 10.1101/299537.

[62] Oksanen JF, Blanchet G, Friendly M, Kindt R, Legendre P, McGlinn D (2018). vegan: Community Ecology Package. R package version 2.5–3.

[63] Bray JR, Curtis JT (1957). An ordination of the upland forest community of southern Wisconsin. Ecol Monogr.

