# Supplementary figures and images for: Integrated genome-wide investigations of the housefly, a global vector of diseases reveal unique dispersal patterns and bacterial communities across farms
Source: BMC Genomics. 2020 Jan 21;21:66. doi: 10.1186/s12864-020-6445-z (PMC6975039; doi:10.1186/s12864-020-6445-z)

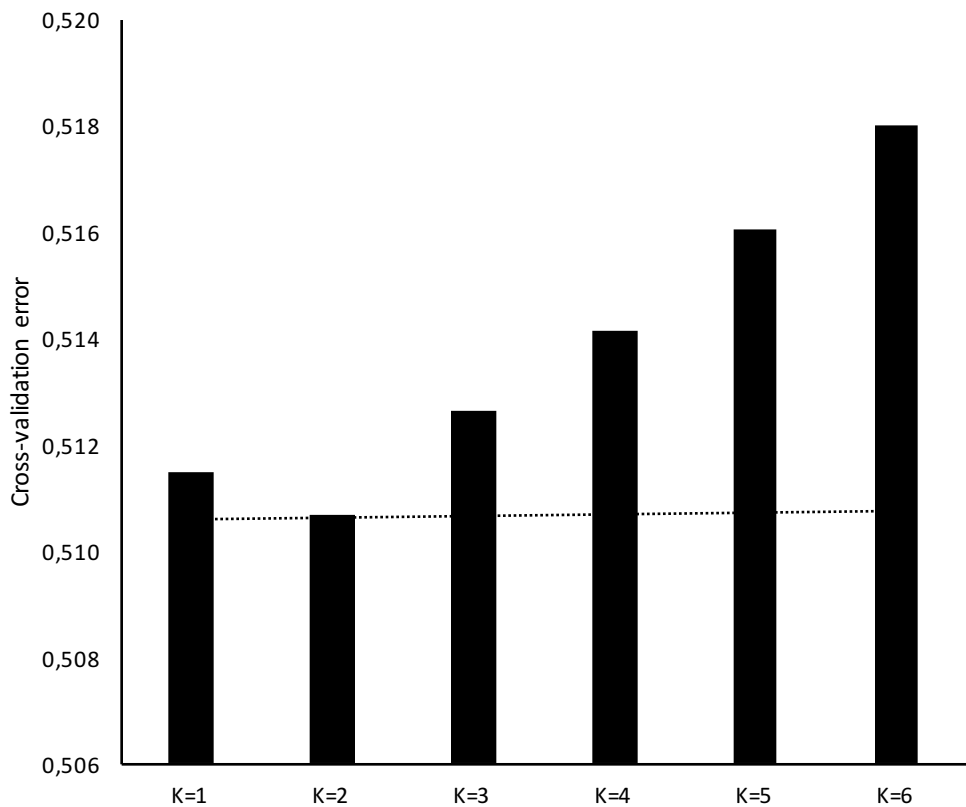

Supplement: Supplementary file 1 — Additional file 1 : Figure S1. ADMIXTURE cross-validation error results. Plot of ADMIXTURE cross-validation error from K=1 through K=6 for both timepoints and sexes. Analysis with K = 2 gave the lowest cross-validation error. [file 12864_2020_6445_MOESM1_ESM.pdf]

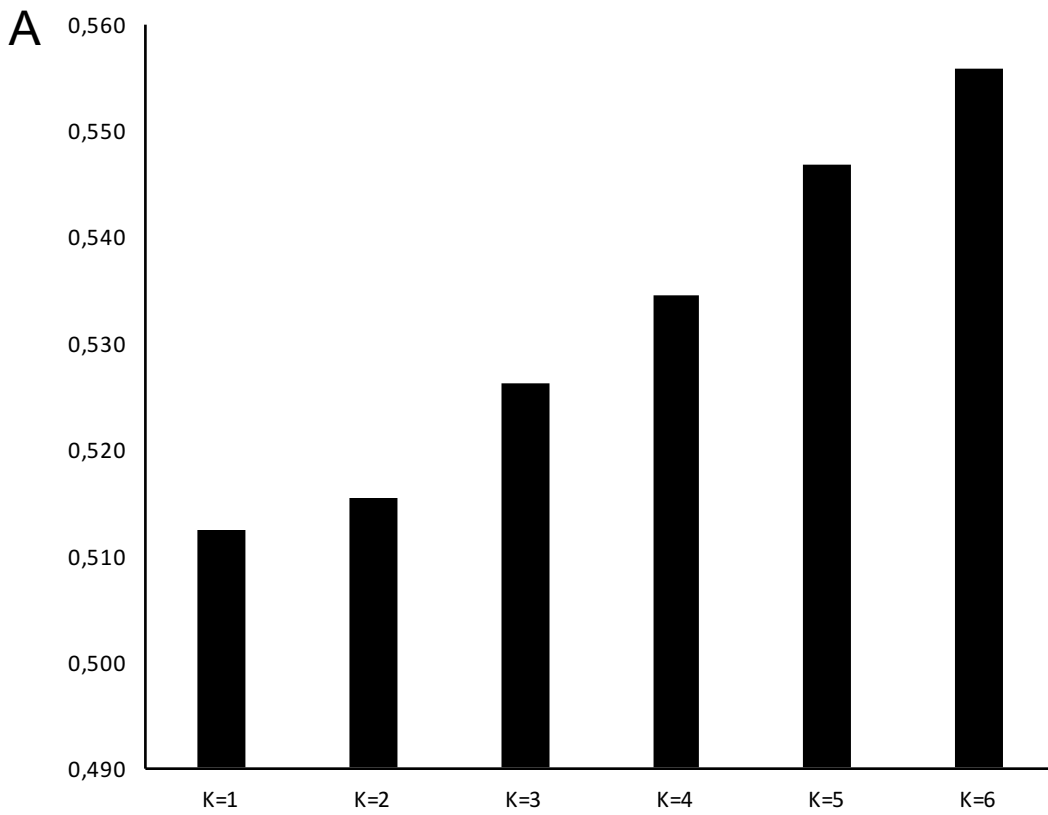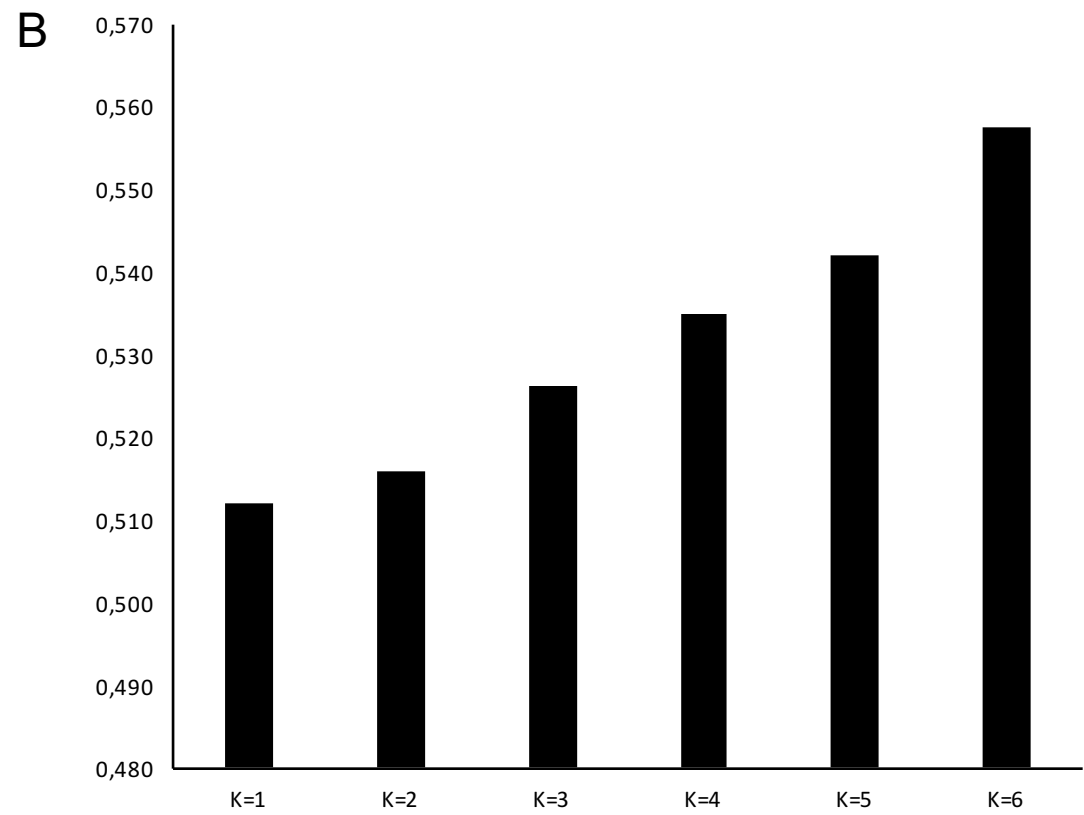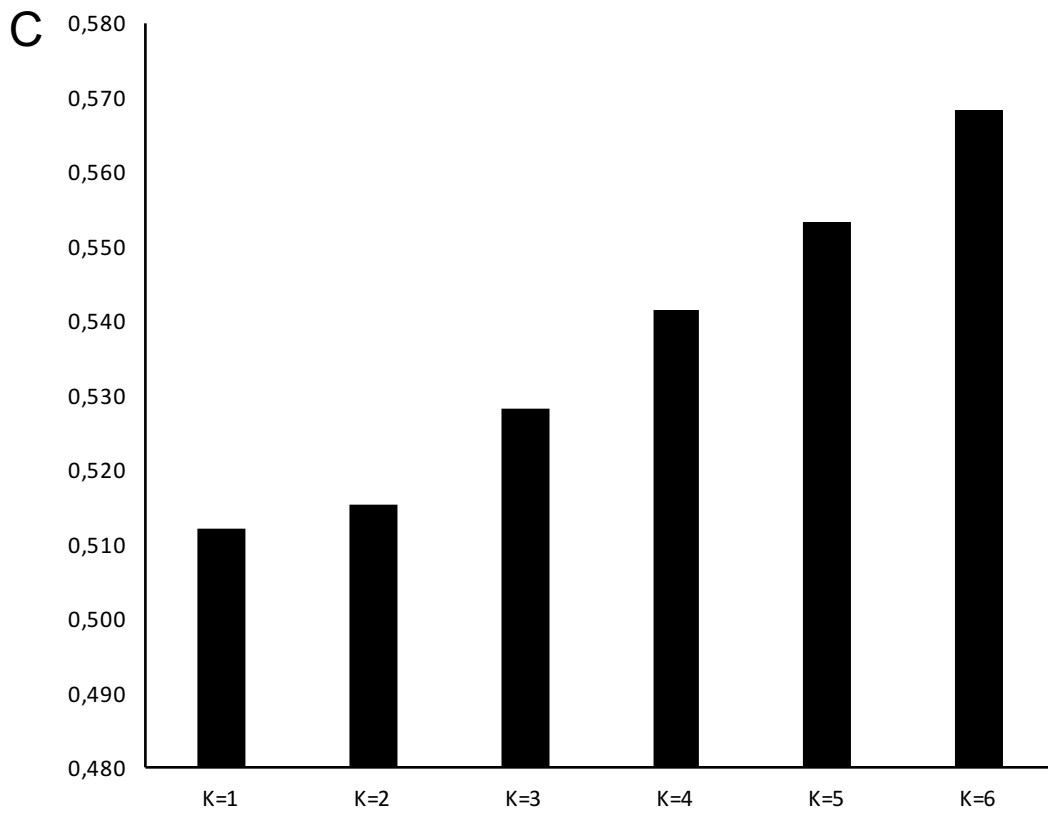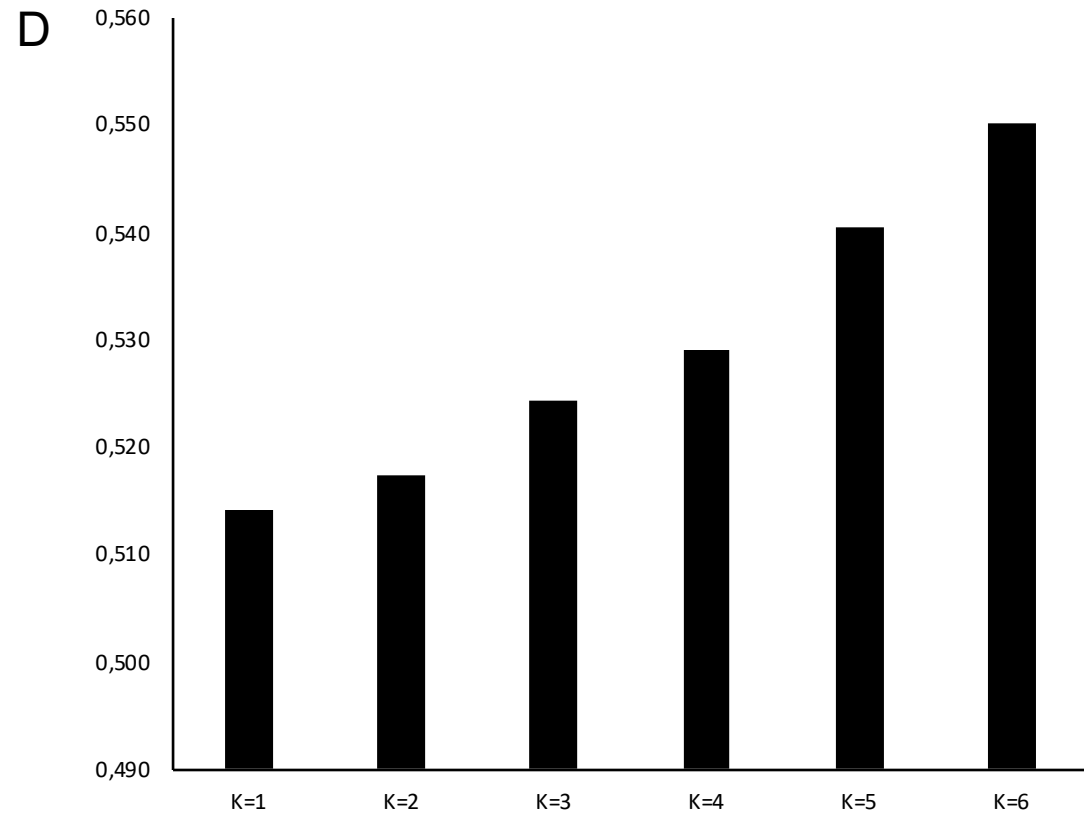

Supplement: Supplementary file 2 — Additional file 2 : Figure S2. ADMIXTURE cross-validation error results. Plots of ADMIXTURE cross-validation error from K=1 through K=6 for males collected in early summer (a), females collected in early summer (b), males collected in late summer (c), and females collected in late summer (d). Analysis with k = 1 gave the lowest cross-validation error in all cases. [file 12864_2020_6445_MOESM2_ESM.pdf]

Cumulative read abundance(%)

100

75

50

25

0

1

10

100

1000

Rank abundance

Population

1

2

3

4

5

6

8

9

10

11

12

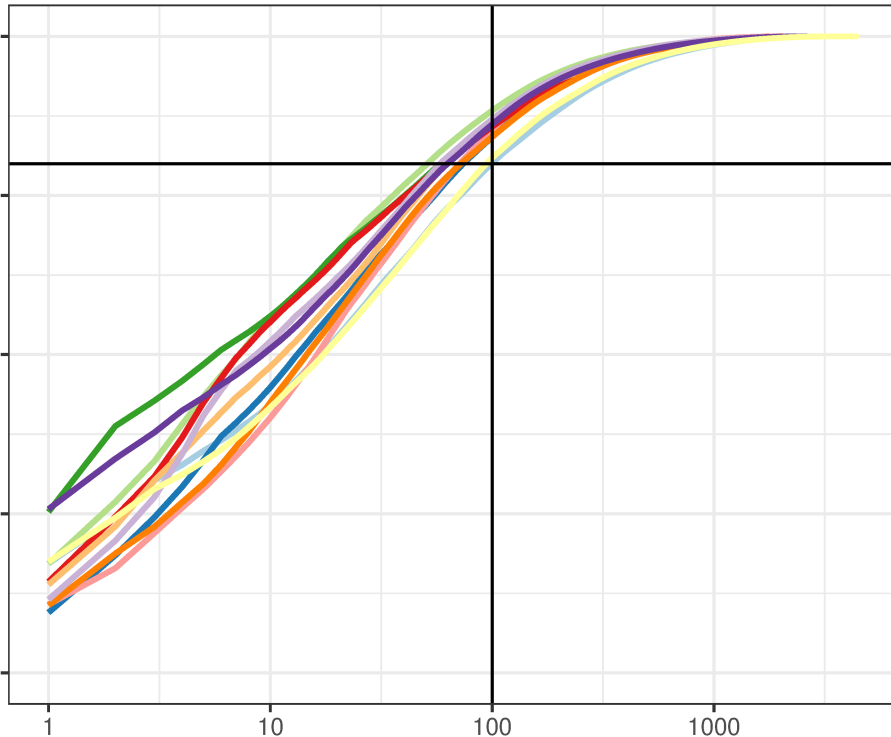

Supplement: Supplementary file 3 — Additional file 3 : Figure S3. Rank abundance curve. Rank abundance curve of sequencing data generated using 432 houseflies. A line is drawn at 100 OTUs, representing 80% of total read abundance. [file 12864_2020_6445_MOESM3_ESM.pdf]

A: House Fly Opt. ApeKI

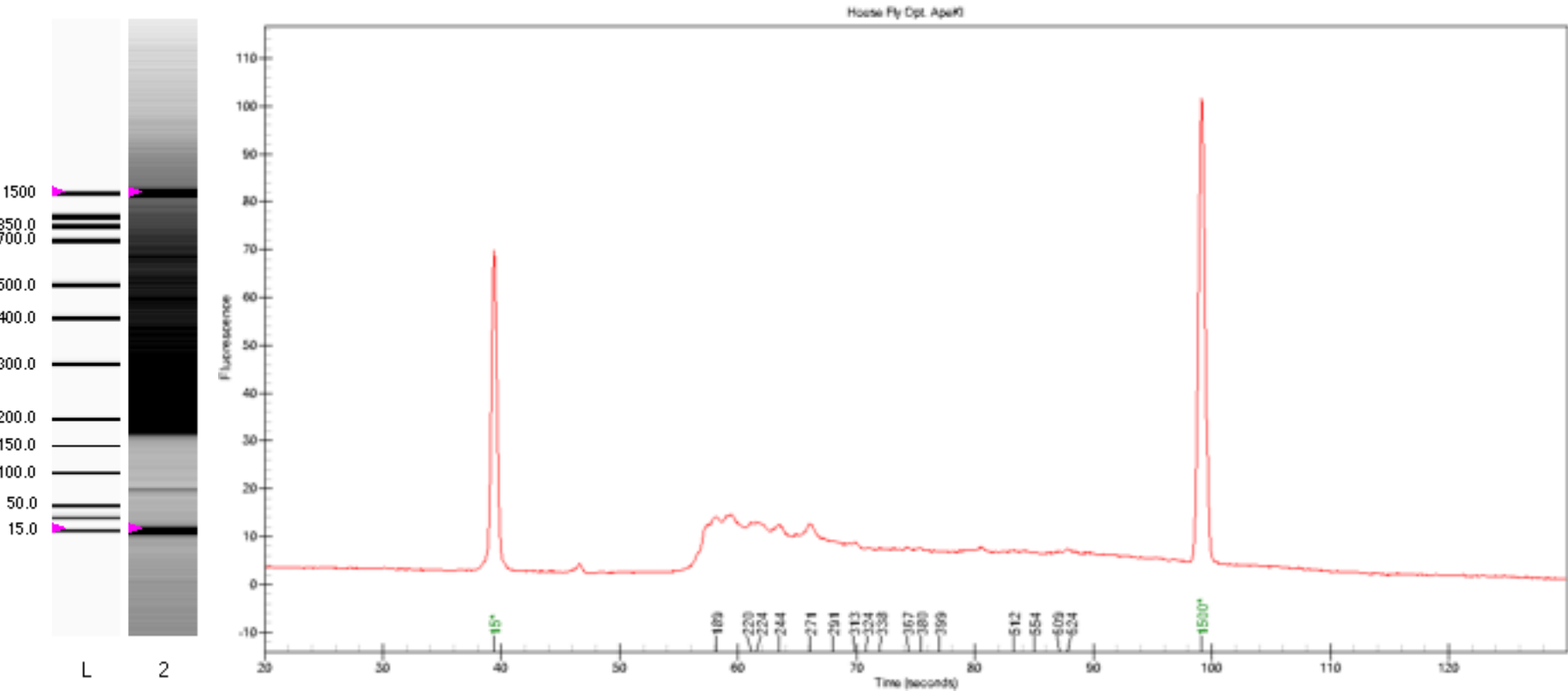

B: House Fly Opt. EcoT22I

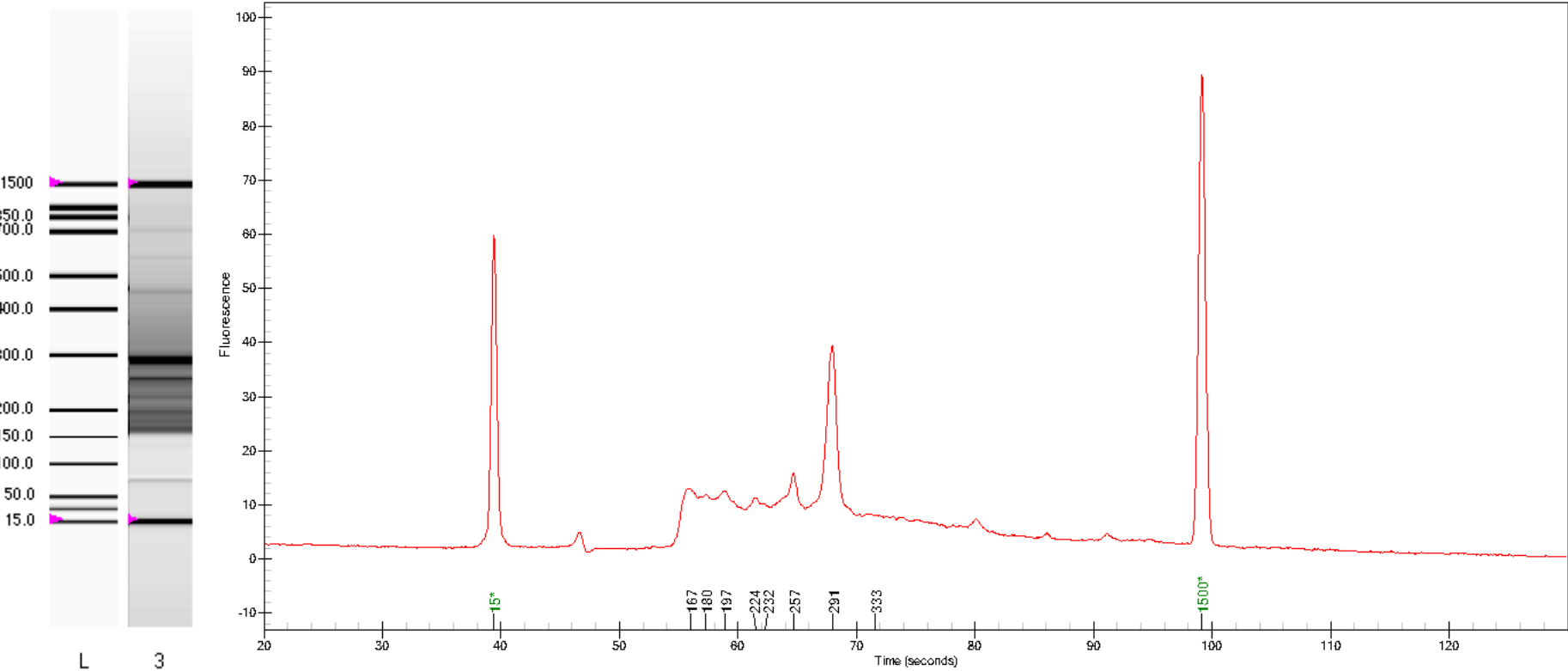

C: House Fly Opt. PstI

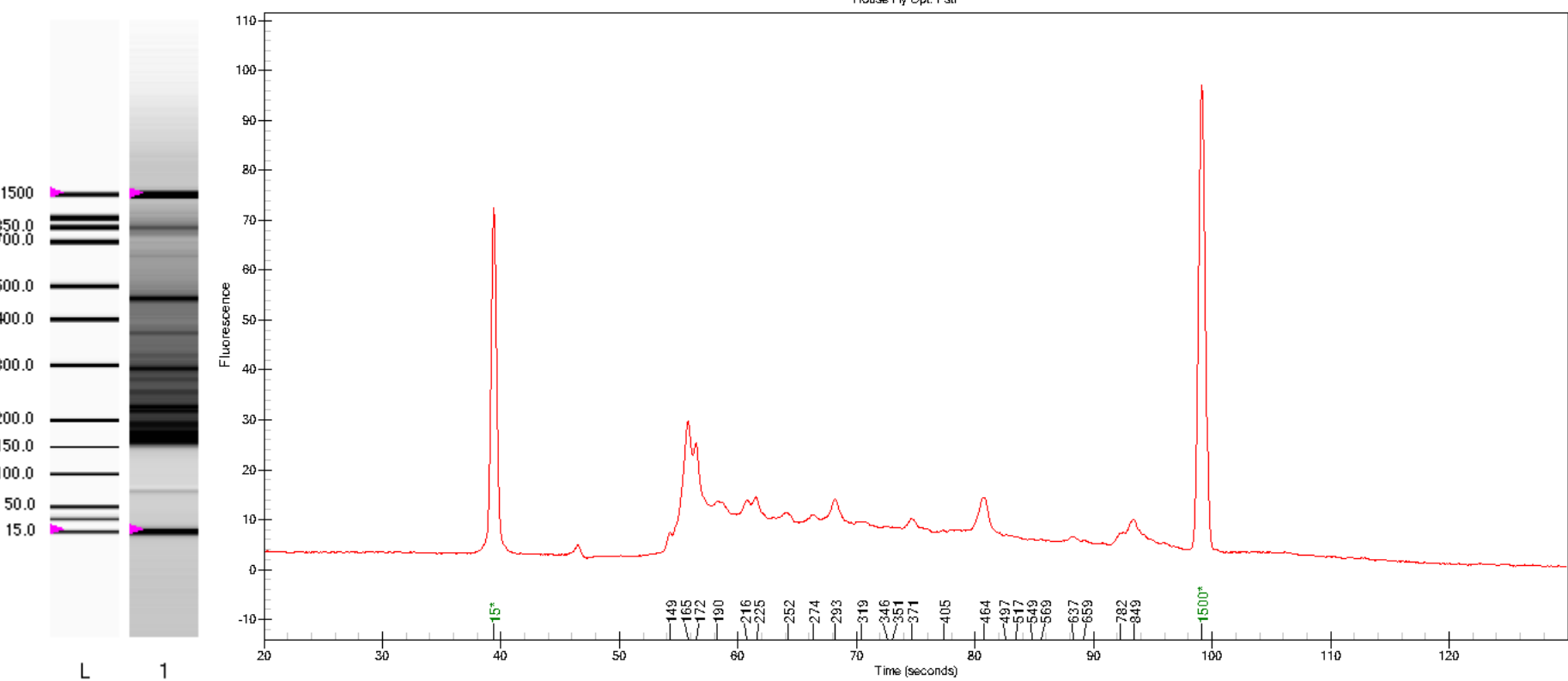

Supplement: Supplementary file 5 — Additional file 5 : Figure S5. Fragment size distribution. Fragment size distribution of GBS libraries made with a single DNA sample using a) ApeKI; b) EcoT22I; and c) PstI restriction enzymes. The x-axis represents elution time and the y-axis shows fluorescence units. Numbers below hatch marks on the x-axis indicate fragment size (bp). Peaks at 15 and 1500 bp are size standards. [file 12864_2020_6445_MOESM5_ESM.pdf]
