# Supplementary material for: Integrated genome-wide investigations of the housefly, a global vector of diseases reveal unique dispersal patterns and bacterial communities across farms
Source: BMC Genomics. 2020 Jan 21;21:66. doi: 10.1186/s12864-020-6445-z (PMC6975039; doi:10.1186/s12864-020-6445-z)
Supplement: Supplementary file 4 — Additional file 4 : Figure S4. Heatmap of abundantly observed OTUs. Microbial community composition. Heatmap of the 50 most abundantly observed OTUs in 11 sampled populations, sorted by season, then by sex. The highest possible taxonomic classification is displayed for each OUT. [file 12864_2020_6445_MOESM4_ESM.pdf]

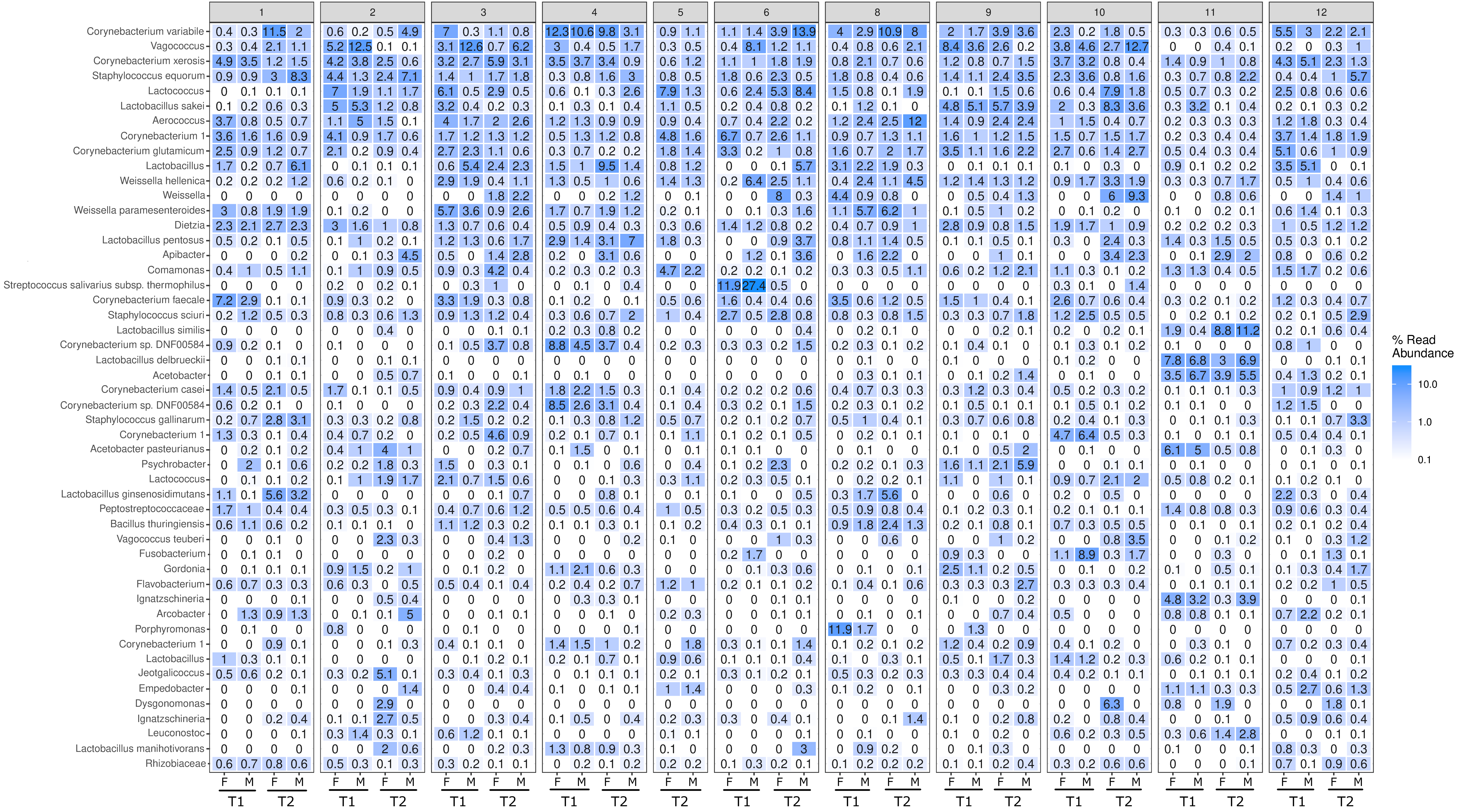

1
2
3
4
5
6
8
9
10
11
12
Corynebacterium variabile
Vagococcus
Corynebacterium xerosis
Staphylococcus equorum
Lactococcus
Lactobacillus sakei
Aerococcus
Corynebacterium 1
Corynebacterium glutamicum
Lactobacillus
Weissella hellenica
Weissella
Weissella paramesenteroides
Dietzia
Lactobacillus pentosus
Apibacter
Comamonas
Streptococcus salivarius subsp. thermophilus
Corynebacterium faecale
Staphylococcus sciuri
Lactobacillus similis
Corynebacterium sp. DNF00584
Lactobacillus delbrueckii
Acetobacter
Corynebacterium casei
Corynebacterium sp. DNF00584
Staphylococcus gallinarum
Corynebacterium 1
Acetobacter pasteurianus
Psychrobacter
Lactococcus
Lactobacillus ginsenosidimutans
Peptostreptococcaceae
Bacillus thuringiensis
Vagococcus teuberi
Fusobacterium
Gordonia
Flavobacterium
Ignatzschineria
Arcobacter
Porphyromonas
Corynebacterium 1
Lactobacillus
Jeotgalicoccus
Empedobacter
Dysgonomonas
Ignatzschineria
Leuconostoc
Lactobacillus manihotivorans
Rhizobiaceae
F M F M
T1 T2
% Read Abundance
10.0
1.0
0.1
